# Recommendations for the diagnosis and management of childhood Prader-Willi syndrome in China

**DOI:** 10.1186/s13023-022-02302-z

**Published:** 2022-06-13

**Authors:** Dai Yang-Li, Luo Fei-Hong, Zhang Hui-Wen, Ma Ming-Sheng, Luo Xiao-Ping, Liu Li, Wang Yi, Zhou Qing, Jiang Yong-Hui, Zou Chao-Chun, PWS Cooperation Group of Rare Diseases Branch of Chinese Pediatric Society, Shan Xiao-Ou, Yang Yu, Zhang Hui-Feng, Tian Zhi-Liang, Sun Bo, Lu Mei, Cheng Ya-Ying, Yang Ying, Yu Xiong-Ying, Zhang Jing, Chen Xiao-Hong, Yang Fan, Ma Hong-Wei, MireguIi Maimaiti, Zhang Gai-Xiu, Chen Xiao-Hong, Li Gui-Mie, Tong Fan, Zhejiang Expert Group for PWS, Zhi Ming-Qiang, Zhou Qiong, Gao Yuan, Wang Kan, Ying Xiao-Ming, Zhang Jian-Ping, Wang Chun-Lin, Jiang Chun-Ming, Xiao Rui

**Affiliations:** 1 Children’s Hospital of Zhejiang University School of Medicine, No. 3333 Binsheng Road, Hangzhou, 310003 China; 2 Children’s Hospital of Fudan University, Shanghai, China; 3 Xinhua Hospital Affiliated to Shanghai Jiao Tong University School of Medicine, Shanghai, China; 4 Peking Union Medical College Hospital, Beijing, China; 5 Tongji Hospital, Tongji Medical College of HUST, Wuhan, China; 6 Guangzhou Women and Children’s Medical Center, Guangzhou, China; 7 Fujian Children’s Hospital, Fuzhou, China; 8 Yale University, 69 Lexington Gardens, Northern Haven, CT 06473 USA

**Keywords:** Prader-Willi syndrome, Diagnosis, Management, Guidelines, Child, China

## Abstract

Prader-Willi syndrome (PWS) is a complex and multisystem neurobehavioral disease, which is caused by the lack of expression of paternally inherited imprinted genes on chromosome15q11.2-q13.1. The clinical manifestations of PWS vary with age. It is characterized by severe hypotonia with poor suck and feeding difficulties in the early infancy, followed by overeating in late infancy or early childhood and progressive development of morbid obesity unless the diet is externally controlled. Compared to Western PWS patients, Chinese patients have a higher ratio of deletion type. Although some rare disease networks, including PWS Cooperation Group of Rare Diseases Branch of Chinese Pediatric Society, Zhejiang Expert Group for PWS, were established recently, misdiagnosis, missed diagnosis and inappropriate intervention were usually noted in China. Therefore, there is an urgent need for an integrated multidisciplinary approach to facilitate early diagnosis and optimize management to improve quality of life, prevent complications, and prolong life expectancy. Our purpose is to evaluate the current literature and evidences on diagnosis and management of PWS in order to provide evidence-based guidelines for this disease, specially from China.

## Introduction

Prader-Willi syndrome (PWS, ORPHA:739) is a complex and multisystem neurobehavioral disorder, which is caused by the lack of expression of paternally inherited imprinted genes on chromosome15q11.2-q13.1 [1, 2]. Down JL. first described the clinical manifestations of PWS in an adolescent female in 1887 [3]. However, this syndrome was not recognized until 1956, when Prader A. and Willi H. reported nine individuals with similar clinical findings [4]. Its estimated incidence ranges from 1 in 10,000 to 1 in 30,000, with equal number of males and females affected [5]. The clinical features of PWS vary with age. It is characterized by severe hypotonia with poor suck and feeding difficulties in the early infancy [6], followed by overeating in late infancy or early childhood and progressive development of morbid obesity unless the diet is externally controlled. Motor milestones and language development are delayed, and all individuals have some degree of cognitive impairment. A distinctive behavioral phenotype is common, with temper tantrums, stubbornness, and manipulative and compulsive behaviors. Hypogonadism occurs in both males and females and manifests as genital hypoplasia, underdevelopment of puberty, and, in most cases, infertility. Short stature is common and associated with growth hormone (GH) deficiency (GHD). Typical facial features, strabismus and scoliosis are common, and there is an increased incidence of sleep disorders and type 2 diabetes mellitus, especially in obese patients [7, 8]. PWS is the most common genetic cause of severe obesity in children. Recently, some rare disease networks, including PWS Cooperation Group of Rare Diseases Branch of Chinese Pediatric Society, Zhejiang Expert Group for PWS, were established. Early diagnosis and treatment can significantly improve the prognosis. However, misdiagnosis, missed diagnosis and inappropriate intervention were usually noted. Therefore, there is an urgent need for an integrated multidisciplinary approach to facilitate early diagnosis and optimize management to improve quality of life, prevent complications, and prolong life expectancy. Our purpose is to evaluate the current literature and evidences on diagnosis and management of PWS in order to provide evidence-based guidelines for this disease.

## Clinical characteristics

### Clinical features

The clinical features of PWS vary with age. It is characterized by severe hypotonia with poor suck and feeding difficulties in the early infancy [6, 9, 10], followed by polyphagia in late infancy or early childhood and progressive development of morbid abdominal obesity unless the diet is externally controlled. The main clinical manifestations of perinatal period are decreased fetal movement, polyhydramnios, breech presentation and non-term delivery. While in infancy, PWS is characterized by hypotonia, feeding problems with a poor sucking reflex, central sleep apnea [11], hypotonia, motor delays and temperature instability [12].

The characteristic facial features of PWS include dolichocephaly or a narrow head shape, a narrow bifrontal diameter, strabismus, almond-shaped palpebral fissure, small upturned nose, down-turned corners of the mouth with xerostonia and abnormality of the dentition, hypopigmentation of the skin comparing with other family members. Physical manifestations include hypopigmentation of the skin and hair, hypogonadism with clitoral hypoplasia and hypoplastic labia in females and a small penis and cryptorchidism in males, short stature, small hands and short feet, and motor delay [13].

In early childhood (about 2–6 years of age), additional features appear including temper tantrums, food seeking behavior and polyphagia leading to abdominal obesity, if the diet is not externally controlled. In addition, intellectual disabilities, specific learning disabilities, behavioral abnormalities including repetitions, compulsions, emotional outbursts and skin picking complicated by a high pain threshold, also develop during childhood [14–16]. A study of 31 Chinese patients with PWS suggested that there was a lower incidence of short stature in Chinese patients with PWS than that in other studies [17, 18], although none of the participants was treated with growth hormone, which may be one of the unique features of Chinese PWS population [19]. Additionally, it was also reported that dysmorphic face, and skin picking lesions are less common among Chinese patients with PWS than that in Western patients with PWS [19].

### Genotype

Genetically, PWS is an imprinted disease owing to the lack of expression of genes inherited from the paternal chromosome 15q11.2-q13.1 region. Our understanding of the molecular basis of PWS has changed dramatically with molecular genetic studies. The most common molecular mechanism of PWS is found in about 65%-75% of cases who show a de novo typical deletion on the paternal chromosome 15q11.2-q13.1 region, including two main subtypes. Subtype Ia deletion is larger, involving chromosome 15q proximal breakpoint (BP), BPI, to a distal breakpoint, BPIII. Subtype Ib deletion is smaller, involving BPII to BP III [20]. Comparing to the typical subtype Ia or Ib deletion, some atypical cases show smaller or greater deletion in size. Very few can be classified as subtype Ic (BP I-BP IV) and subtype Id (BP I-BP V) [2, 21–23]. An unusual or atypical deletion is seen in about 5% of PWS individuals. A small microdeletion (about 118 Kb) within the interval between *SNRPN* and *UBE3A* that spans the SNORD116 clusters and its host transcripts is also an etiological factor in PWS [24, 25]. The second most common molecular mechanism of PWS is found in nearly 20%-30% of cases and associated with maternal uniparental disomy (mUPD) 15, in which both copies of chromosome 15 come from the mother. It includes two subtypes. Subtype IIa is isodisomy in which both chromosome 15 come from the grandmother or grandfather. While the subtype IIb is heterodisomy in which one chromosome 15 is from the grandmother and the other is from the grandfather. Due to the imprinted gene regulation, the same genes in the maternal chromosome, 15q11.2-q13.1, are intact in structure but repressed at the transcriptional level because of the epigenetic mechanism, which is primarily by methylation [26]. The third molecular mechanism of PWS is less common and is found in about 3% patients with PWS, termed imprinting defect (ID), including epimutation and the PWS imprinting center (PWS-IC) deletion. The epigenotype measured by DNA methylation for both chromosomes in the 15q11.2-q13.1 region is maternal. Some other rare genetic alterations and molecular mechanisms of PWS have been reported, such as Robertsonian translocation (15;15) inherited from the mother. Except for the pathogenic variants in the *MAGEL2* gene that have been associated with Schaaf-Yang syndrome [27], no pathogenic variants of *SNRPN* was reported in PWS patients [28, 29] as showed in Table 1. It was reported that there was a higher incidence of paternal deletion in Chinese patients with PWS than that in Western patients with PWS and there was also a correspondingly lower incidence of mUPD [19], which were consistent with those of other Asian studies [17, 30, 31].On rare occasions, a second chromosomal anomaly may be found in addition to the 15q11.2 deletion, such as Klinefelter syndrome [32–35]. Therefore, counseling families about the clinical prognosis of the proband can be affected by the presence of additional chromosomal abnormalities.

**Table 1:** The genotype of Prader-Willi syndrome

Genotypes	Subtypes	Molecular mechanism
Paternal deletion (I)	Ia	BP I-BP III
	Ib	BP II-BP III
	Ic	BP I-BP IV
	Id	BP I-BP V
mUPD (II)	IIa	Isodisomy
	IIb	Heterodisomy
Imprinting defect (III)	IIIa	Imprinting center deletion
	IIIb	Epimutation
Others (e.g. Robertsonian translocation)		Robertsonian translocation [15] from mother

BP, breakpoint; ID: imprinting defect; mUPD: maternal uniparental disomy

### Genotype–phenotype correlations

Although it was reported that there were differences in the frequency or severity of certain features among different genotypes, mainly in the paternal deletion and mUPD 15, there was no specific phenotypic feature which was known to be associated with either of the 2 main genotypes. It was reported that PWS patients with a paternal deletion had more prominent feeding problems, speech articulation impairment and sleep disturbance [36, 37]. A study of 31 Chinese patients with PWS showed that small hands and feet were more common in patients with paternal deletions than that in patients with mUPD. Besides, patients with paternal deletions were also more likely to be associated with hyperphagia and excessive weight gain than that patients with mUPD [19]. Additionally, there were differences between the paternal deletion and mUPD 15 in maladaptive behaviors. Compared with the mUPD 15, the paternal deletion type had higher self-injury and stealing scores [38]. While individuals with mUPD 15 had a slightly higher verbal IQ and milder behavior problems [39, 40]. However, it was reported that patients with mUPD 15 were at greater risk of having a psychosis in adulthood [41–44] and autism spectrum disorder (ASD) [45–48]. In one study, compared to 47% with a paternal deletion, as many as 74% individuals with mUPD 15 had been prescribed psychotropic medication [14]. However, there were also some reports of no difference between the two genotypes.

## Diagnosis

The proposed clinical diagnostic criteria have changed over the past few decades [18]. A consensus was established in 1993 when genetic testing was very limited and updated in 2001 [18, 49]. The age of diagnosis has dropped significantly over the past decade, with most cases now being diagnosed in the first few months of life [50]. This allows the earlier introduction of treatments in order to reduce the morbidity particularly by preventing obesity, which will not only improve the quality of life of patients with PWS, but also reduce the burden on families and caregivers [51, 52]. The diagnosis of PWS relies on a combination of clinical features and genetic analysis [10, 13].

### Consensus diagnostic criteria

Consensus clinical diagnostic criteria for PWS using a numerical scale was developed in 1993 before the availability of diagnostic testing and later revised with updates based on clinical findings and presentation in 2001 [18, 49], which included 8 major criteria. while in 2012, Cassidy SB changed it to 6 [5]. To score, major criteria are weighted at 1 point each, and minor criteria are weighted at 1⁄2 point each. Supportive findings increase the certainty of diagnosis but are not scored. It requires 5 points (at least 4 of them major) for children 3 years of age or younger and 8 points (at least 5 of them major) for children 3 years of age or older, as showed in Table 2. However, confirmation of the diagnosis of PWS requires molecular genetic testing [53].

**Table 2:** Consensus diagnostic criteria for Prader-Willi syndrome

*Major criteria*	
1	Neonatal and infantile central hypotonia with poor suck, gradually improving with age
2	Feeding problems in infancy with need for special feeding techniques and poor weight gain/failure to thrive
3	Excessive or rapid weight gain on weight-for-length chart (excessive is defined as crossing two centile channels) after 12 months but before 6 years of age; central obesity in the absence of intervention
4	Characteristic facial features with dolichocephaly in infancy, narrow face or bifrontal diameter, almond-shaped eyes, small-appearing mouth with thin upper lip, down-turned corners of the mouth (3 or more are required)
5	Hypogonadism-with any of the following, depending on age
	(1) Genital hypoplasia, (male: scrotal hypoplasia, cryptorchidism, small penis and/or testes for age (5th percentile); female: absence or severe hypoplasia or labia minora and/or clitoris
	(2) Delayed or incomplete gonadal maturation with delayed pubertal signs in the absence of intervention after 16 years of age (male: small gonads, decreased facial and body hair, lack of voice change; female: amenorrhea/oligomenorrhea after age 16)
6	Global developmental delay in a child 6 years of age; mild to moderate mental retardation or learning problems in older children
*Minor criteria*	
1	Decreased fetal movement or infantile lethargy or weak cry in infancy, improving with age
2	Characteristic behavior problems-temper tantrums, violent outbursts, and obsessive–compulsive behavior; tendency to be argumentative, oppositional, rigid, manipulative possessive, and stubborn; perseverating, stealing, and lying (5 or more of these symptoms required)
3	Sleep disturbance and sleep apnea
4	Short stature for genetic background by age 15 (in the absence of growth hormone intervention)
5	Hypopigmentation-fair skin and hair compared with family
6	Small hands (25th percentile) and/or feet (10th percentile) for height age
7	Narrow hands with straight ulnar borders
8	Eye abnormalities (esotropia, myopia)
9	Thick viscous saliva with crusting at corners of the mouth
10	Speech articulation defects
11	Skin picking
*Supportive findings*	
1	High pain threshold
2	Decreased vomiting
3	Temperature instability in infancy or altered temperature sensitivity in older children and adults
4	Scoliosis and/or kyphosis
5	Early adrenarche
6	Osteoporosis
7	Unusual skill with jigsaw puzzles
8	Normal neuromuscular studies

To score, major criteria are weighted at 1 point each, and minor criteria are weighted at 1⁄2 point each. Supportive findings increase the certainty of diagnosis but are not scored. Clinical diagnosis requires 5 points (at least 4 of them major) at age < 3 years; 8 points (at least 5 of them major) at age 3 years or older

### Genetic testing methods

There are various methods to identify genetic alterations in PWS patients using peripheral blood lymphocytes [54, 55]. The DNA methylation pattern of the promoter-exon 1 region of the *SNURF*-*SNRPN* bicistronic gene (15q11.2) was used as the most sensitive laboratory method for the diagnosis of PWS [56–58]. Options for DNA methylation analysis include methylation-specific polymerase chain reaction (MS-PCR) and methylation-specific multiplex ligation-dependent probe amplification (MS-MLPA) [59, 60]. Further approaches can be performed to identify the genetic types and allow appropriate genetic counseling, particularly for the recurrence risk. High-resolution chromosomal microarray analysis (CMA) could be used to determine the presence and size of a chromosome deletion as well as to identify partial mUPD 15 subtype [22]. Fluorescence in-situ hybridization (FISH) with high resolution karyotype may identify the deletion status or rule out a chromosomal rearrangement. However it is used less often nowadays. Although DNA sequencing may detect microdeletion, DNA sequence analysis can be considered for rare situations [61, 62], as showed in Table 3. DNA polymorphism or linkage analysis can be performed to determine whether the inheritance pattern is biparental (normal) or maternal-only (maternal disomy).

**Table 3:** Genetic testing used in Prader-Willi syndrome

Methods	Genotype identified	Uses and limitations
MS-MLPA	Paternal deletion, mUPD, ID, Robertsonian translocation	It can identify > 99% of PWS and can distinguish deletion from other types, but cannot generally distinguish mUPD from an ID (IC deletion and epimutation), unless in rare individuals, a microdeletion of the IC is seen. It also can estimate the size and distinguish most the paternal deletion subtype
MS-PCR	Paternal deletion, mUPD, ID, Robertsonian translocation	It can identify > 99% of PWS, but it cannot distinguish molecular type. It can’t identify IC deletion and key gene pathogenic variant
CMA-SNP array	Paternal deletion, partial mUPD (Isodisomy),	It can identify 80%-90% of PWS and provide information regarding deletions and duplications in the entire chromosome. However, it cannot distinguish the PWS from AS alone. It cannot identify partial mUPD (heterodisomy), ID, Robertsonian translocation and chromosomal rearrangements
FISH	Paternal deletion, Robertsonian translocation	It can identify 65%–75% of PWS, and distinguish paternal deletions from chromosomal rearrangements (e.g. Robertsonian translocation). It may be used for patient’s parents to identify translocation. However, it cannot distinguish normal, mUPD, and ID, and requires living cells
DNA sequence	IC deletion, pathogenic variant, most paternal deletion	It cannot identify mUPD and epimutation. It can be considered for rare situations after DNA methylation analysis, FISH (no deletion), quantitative microsphere hybridization
High-resolution karyotype	Partial paternal deletions, Robertsonian translocation	It may detect most deletions, but requires experienced technician. It should not be used alone because it will miss some deletions, mUPD and ID

AS, Angelman syndrome; CMA, chromosomal microarray; FISH, fluorescence in situ hybridization; IC, imprinting center; ID, imprinting defect; MS-MLPA, methylation-specific multiplex ligation-dependent probe amplification; MS-PCR, methylation-specific polymerase chain reaction; PWS, Prader-Willi syndrome; SNP, single nucleotide polymorphism; mUPD, maternal uniparental disomy

### Procedure of molecular diagnosis

**Fig. 1:**
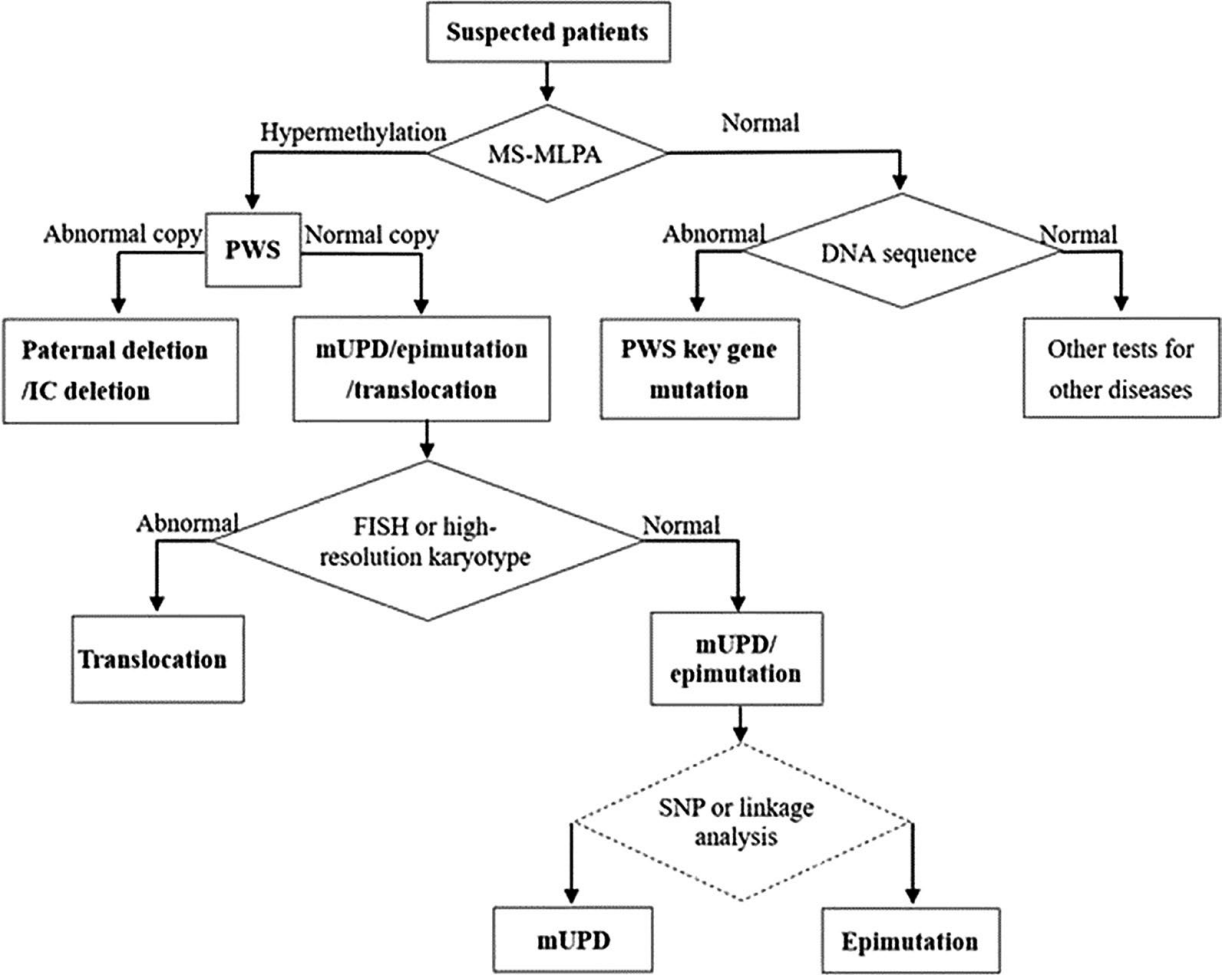
Algorithm for genetic testing for Prader-Willi syndrome. MS-MLPA, methylation-specific-multiplex ligation probe amplification; FISH, fluorescence in situ hybridization; IC, imprinting center; SNP, single nucleotide polymorphism; mUPD, maternal uniparental disomy

DNA methylation analysis is the preferred method for the diagnosis of PWS, which detects more than 99% of the cases, including deletions, mUPD and IC defects (Fig. 1). Common options for DNA methylation analysis include MS-PCR and MS-MLPA. Compared with MS-PCR, MS-MLPA has the advantage of identifying the DNA methylation status as well as deletions. Therefore, MS-MLPA is preferred for the molecular diagnosis of PWS. Hypermethylation with copy number loss implies a paternal deletion while hypermethylation with normal copy number implies the mUPD, epimutation or Robertsonian translocation. FISH or high-resolution karyotype can be performed to distinguish translocation from mUPD and epimutation, if necessary. If the result of FISH is normal, then the DNA polymorphism or linkage analysis can be performed to distinguish mUPD from epimutation. If the result of MS-MLPA is normal, DNA sequence analysis can be performed to identify IC deletion and key genes pathogenic variants for patients highly suspected for PWS. If the result of the DNA sequence analysis is also normal, then additional tests are necessary to perform for further diagnosis.

MS-PCR can be performed first if MS-MLPA is not available [63]. If the result of MS-PCR is abnormal, then CMA with single nucleotide polymorphism (SNP) and copy number variant (CNV) probes can be used to determine the copy number, which may distinguish the deletion type from the other subtypes (e.g. mUPD, epimutation) [2, 59, 60, 64]. If the result of MS-PCR is normal, DNA sequence analysis may also need to identify IC deletion and key genes pathogenic variants. Furthermore, when testing for PWS does not explain all the features, additional tests are also in need to perform for further diagnosis.

After a molecular diagnosis is determined for an affected child, it is highly recommended to test the asymptomatic parents in order to determine the origin of the genetic alteration and the recurrence risk for the purpose of genetic counseling. Although majority of PWS have a low recurrence risk (< 1%), some genetic etiology could lead to high recurrence risk (> 50%), such as paternal balanced translocations or maternal 15/15 Robertsonian translocation.

### Prenatal diagnosis and newborn screening

Prenatal diagnosis is not recommended routinely yet, although it can theoretically be suspected in cases of reduced fetal movement and polyhydramnios [6, 65–67]. Genetic testing can be performed on samples obtained from chorionic villus sampling and amniocentesis [68]. FISH or CMA can easily pick up deletions from such tissues [69], but DNA methylation analysis is necessary in cases of mUPD and ID [57]. However, clinical laboratories that perform prenatal DNA methylation analysis are reluctant to use such chorionic villus samples because of the corresponding hypomethylation of the tissue. The introduction of techniques such as comparative array genomic hybridization can be used to prenatal diagnosis of PWS owing to deletions [70].

Although it can theoretically be suspected in cases of hypotonia (for both sexes) and testicular ectopy (in boys), the clinical diagnosis of PWS in newborns is challenging because the distinctive phenotypic characteristics of the disease are not completely evident during this phase. Reliable and low-cost molecular analysis techniques are imperative for accurate and early diagnosis to start precise treatment. It was reported that DNA extracted from dried blood spot (DBS) could be analyzed by the methylation-sensitive high-resolution melting (MS-HRM) methodology, which used a unique pair of primers to amplify the promoter-exon 1 region of the *SNURF*-*SNRPN* locus and reveal its methylation status [57], providing an accurate approach for genetic screening of PWS in newborns [71]. Compared to the traditional whole blood methods, the use of the DBS sample as the main source of DNA provides several advantages: demanding only a small amount of blood, less invasive procedure, ease of storage, and transportation [72]. However, the technology is not widely available.

### Differential diagnosis

Patients with negative testing for PWS should be investigated for other chromosomal deletions and duplications associated with PWS-like characteristics [27, 73], as well as possible single-gene defects [74–77].

Hypotonia in infancy is seen in many other conditions [6, 78, 79]. The common causes are neonatal sepsis and central nervous system depression. Congenital myotonic dystrophy type I is characterized by hypotonia and severe generalized weakness at birth, often accompanied by respiratory insufficiency, developmental delay, and early death [79, 80]. Additionally, a lot of muscular and neurological diseases are characterized by neonatal hypotonia, including some spinal muscular atrophy. In these cases, there may be shortness of breath, which is rarely seen in patients with PWS. Molecular genetic testing, electromyography, and/or muscle biopsy are usually required to differentiate these diseases. Many congenital metabolic errors may also manifest as hypotonia and are found to be lethargy or non-lethargy in infancy. Pompe disease is in particular worth considering [81].

Several genetic disorders are characterized by obesity, developmental delay, intellectual disability with or without hypogonadism. Angelman syndrome (AS) and Fragile X syndrome can both include obesity in a subset of individuals but no hypogonadism [82, 83]. mUPD for chromosome 14 causes early motor and speech delay, excess weight, hypotonia, and can also present with feeding problems, short stature, small hands and feet, and scoliosis [84, 85]. However, the typical clinical presentation of it is early puberty and joint laxity.

Many cytogenetic abnormalities result in overlapping manifestations with PWS, such as deletion of 1p36, 2q37.3, 6q16.2, and 10q26 and duplication of 3p25.3.26.2 and Xq27.2-ter [75, 86–89]. Therefore, when testing for PWS is negative or does not explain all the features, CMA is an appropriate test. Careful clinical evaluation by a medical geneticist or other trained diagnostician is highly recommended to direct testing appropriately and may avoid the unnecessary expense of molecular testing for diagnoses which are less likely based on clinical findings.

## Management

Management of the manifestations of PWS depends on age and should include guidance to manage the consequences and expectations of the syndrome. Multidisciplinary treatment has been shown to reduce hospital stays and prevent early obesity.

### Focus on different times

In infancy, in order to assure adequate nutrition, special nipples or gavage feeding is usually needed because poor suck will lead to developmental delay if they are untreated. Growth measurements including height, weight, and head circumference should be obtained and plotted at diagnosis [90]. The adrenal cortex function should also be noticed. In addition, physical therapy may improve the muscle strength. If individuals have cryptorchidism, hormonal and surgical treatment should be considered.

In childhood (after 1 year of age), guardians should strictly supervise the daily food intake to avoid excessive weight gain and encourage physical activity. Guardians are advised to take an educational plan for their children [91]. Replacement of sex hormones at puberty is beneficial to produce adequate secondary sexual characteristics, and should be supervised by a pediatric endocrinologist. Adrenal function should be reassessed later in the adolescence or adulthood (even if it is normal in infants).

In adulthood, it is also of great importance for PWS individuals to regulate behavior and manage weight to prevent morbid obesity. Involvement of occupational therapy helps with transitioning to independence in areas such as self-care and performing other activities of daily living.

### GH treatment

Recombinant human GH (rhGH) therapy in patients with PWS has been used by the medical community since its approval in the United States in 2000 and in Europe in 2001 [92]. It is beneficial to normalize height, increase lean body mass, flexibility and activity levels, and reduce fat mass [93–99].

No consensus was reached on the age of starting rhGH treatment, although all agreed to the benefits of treating before the onset of obesity. Researches have suggested that the earlier growth hormone therapy is started, the more benefit it has (often around 3–6 month of age) [96, 100, 101]. Before initiation of rhGH therapy, patients with PWS should have a confirmed molecular diagnosis and expert multidisciplinary evaluation. GH stimulation testing should not be required as part of the therapeutic decision-making process in infants and children with PWS [102]. Selection of patients with PWS for rhGH therapy and dosing strategy should not depend on the genetic class of PWS. However, when patients with PWS have one or more of the following conditions: severe obesity, uncontrolled diabetes, untreated severe obstructive sleep apnea [11], active cancer, and active psychosis, the treatment of rhGH should be under careful consideration. Scoliosis should not be considered as a contraindication to rhGH treatment in patients with PWS.

Infants and children with PWS should start with a daily dose of 0.5 mg/m^2^·d with subsequent adjustments toward 1.0 mg/m^2^·d according to clinical response and be guided by maintenance of physiological levels of insulin like growth factor 1 (IGF-1). IGF-1 levels in patients with rhGH treatment should be maintained within the upper part of normal range (maximum + 2SDS) for healthy, age-matched normal individuals. Clinical outcome priorities should vary depending on age and on the presence of physical, mental, and social disability. Treatment with rhGH must be in the context of appropriate dietary, environmental, and lifestyle interventions necessary for care of all patients with PWS. Adults with PWS should receive a starting dose of 0.1–0.2 mg/d based on age, presence of edema, prior rhGH exposure and sensitivity, and concomitant oral estrogen use. Subsequent dosage titration should be based on clinical response, age-, and sex-appropriate IGF-1 levels in the 0 to + 2 SDS range [102, 103].

It seems that GH treatment is generally safe and well tolerated [104]. Thyroid function should be estimated and levothyroxine should be supplied before GH treatment if hypothyroidism was noted. Although GH treatment could result in an increase in fasting plasma glucose levels and insulin resistance, it usually does not increase the onset of diabetes which seems to be most influenced by obesity [103, 105]. Additionally, because there have been reports of accidental deaths in patients with PWS during GH treatment, monitoring for breathing problems and sleep apnea is recommended [106]. Other potential side effects include joint pain, edema, scoliosis and intracranial hypertension [104]. Therefore, it is recommended to perform polysomnography before starting treatment and periodically after, monitoring growth rate, glucose profile, IGF-1 levels, thyroid function, monitoring development and/or progression of scoliosis during growth [107], and liver function (especially for individuals under one year old), as showed in Table 4.

**Table 4:** Evaluation of patients with Prader-Willi syndrome during rhGH treatment

Management monitoring	
Regular clinical assessment of height, weight, growth rate, body composition, pubertal status, scoliosis, IGF-1, thyroid function, and side effects every 3–6 months	
OGTT is recommended to be performed if patients with PWS have history of impaired glucose tolerance, obese, or family history of diabetes	
It had better have an ENT assessment and polysomnography within the first 6 months	
The ENT assessment, polysomnography, and IGF-1 measurement are necessary, if development or worsening of sleep-disordered breathing, snoring, or enlargement of tonsils and adenoids	
If scoliosis is a matter of concern, the X-ray orthopedic assessment can be performed	
Routine measurement of bone age, especially during adolescence	
Monitoring for hypothyroidism	

ENT, ear, nose, and throat; OGTT, oral glucose tolerance test

### Hypogonadism

Unilateral or bilateral cryptorchidism is a common finding in male patients with PWS. Cryptorchidism should be sought and addressed with hormonal and/or surgical treatments. The early treatment of hypogonadism (within the first 6 months of life) is beneficial to many male patients with PWS. The treatment includes human chorionic gonadotropin (hCG) in order to improve phallus size and assist with testicular descent into the scrotal sac in few patients [93]. Surgical correction of cryptorchidism should be completed on the first or at latest in the second year of life [108].

Hypogonadism is present in both males and females and has both a primary and central (hypothalamic) etiology, with the latter believed to be more influential. Although individuals with PWS do enter puberty, the progress is arrested, and puberty is not complete. Treatment of hypogonadism usually commences at around age 11–12 years for females and age 12–13 for males, if it was desired by the parents and child. It is recommended that females can be treated with low-dose estrogen therapy with escalating doses for 2 years or until menarche, at which point they are transitioned to a combined estrogen-progesterone oral contraceptive pill or transdermal patch [5]. The decision to treat hypogonadism in females with PWS is a personal decision for each family and usually relies on the maturity level, independence, and degree of obsessive–compulsive behaviors in PWS patients. Adolescent males with PWS can be treated with either a low-dose transdermal testosterone patch or gel with escalating doses every 3–6 months to allow the testosterone levels to get into the normal range for age or with hCG therapy [109]. Oral testosterone can induce secondary sexual characteristics. GnRH or hCG therapy increases endogenous testosterone production, which can increase testicular volume and lean body mass, but not cause the mood and aggression problems that are characteristic of testosterone therapy [110, 111]. Sex education and contraception should be considered, especially for PWS women, as pregnancy has been reported infrequently [112, 113].

Precocious pubarche is not uncommon in PWS. It is considered to be secondary to obesity and premature adrenarche [114]. Premature adrenarche in PWS is not rapidly progressively or associated with other signs of central precocious puberty, and commonly develops genuinely [114]. Further investigations or treatments are usually not required. Central precocious puberty is very rare in patients with PWS [114].

### Others

At present, the management of PWS is based on symptoms. It was reported that Limosilactobacillus reuteri probiotic could be used to modulate BMI, social behaviors, and gut microbiota in patients with PWS, although further investigation was warranted [115]. Oxytocin (OXT) and its analogue, carbetocin, are extensively studied in PWS clinical trials and have shown potential for treating both hyperphagia and behavior problems in pediatric PWS, although future investigations should confirm the previous study findings with extended follow-up periods within larger, well-defined clinical cohorts and also determine long-term effects and safety [116, 117]. On the basis of a phase 2 clinical trial it is demonstrated that Diazoxide Choline Controlled-Release (DCCR) decreased appetite-related behaviors and fat mass in patients with PWS [118]. Calcium and vitamin D supplements should be considered to optimize attainment of peak bone mass [108]. N-acetylcysteine or topiramate can be used to reduce skin picking [119–121], and recently is reported to be efficient against hyperphagia on Dykens questionnaire (severity and behavior) [122]. Additionally, it was reported that modafinil could be used to treat daytime sleepiness [123]. Although bariatric surgery is currently the most effective therapy to induce weight loss in patients with morbid obesity, its use in PWS remains controversial. It was reported that bariatric surgery could not produce sustainable long-term weight loss or comorbidity resolution in PWS and suggested that bariatric surgery could not be recommended to patients with PWS as a standard treatment [124]. MetAP2 inhibition with beloranib is reported to produce statistically significant and clinically meaningful improvements in hyperphagia-related behaviors and weight loss in participants with PWS [125]. There are also many other new therapies currently in development ranging from very early discovery and preclinical studies to active clinical trials, such as genetic therapies (gene activation by small molecules, CRISPR-based activation, oligonucleotide therapy, AAV-based gene activation, epigenome editing), hyperphagia/obesity drugs (e.g. setmelanotide, tesomet, cannabidivarin, aardvark 101, GLP receptor agonists), devices (e.g. transcranial direct current stimulation) (https://www.fpwr.org/therapeutics-in-development-for-pws).

Routine vaccination is recommended for patients with PWS when their conditions are stable and there are no other contraindications, such as severe malnutrition or infection.

## Genetic counseling

All patients need genetic counseling. It is critical to know the specific genetic etiology in patients with PWS for the appropriate genetic counseling of affected families. Most families have a recurrence risk less than 1%. According to the genetic mechanism and recurrence risk, PWS was divided in 3 types, which is some different from above mentioned genotypes (Table 1). Almost all 15q11.2-q13 deletions are de novo interstitial paternal deletions (Ia), which have a very low recurrence risk (< 1%). A deletion due to chromosomal rearrangement (Ib) has a recurrence risk possibly up to 50%. Therefore, it is necessary to exclude balanced translocation for the father of deletion type PWS patient by FISH, or whole exome sequencing which is still rarely used because of the high cost.

mUPD 15 is typically de novo (IIa), with a recurrence ratio < 1% except if there is a Robertsonian translocation (IIb) mechanism. It is almost 100% recurrence ratio if mother has a 15;15 Robertsonian translocation. Therefore, it is necessary to exclude Robertsonian translocation (15;15) for the mother of PWS by high resolution karyotype or FISH. Rarely, a small marker chromosome is also present in a proband with mUPD 15 [126]. In these instances, it is of great importance to examine both parents’ karyotype because it seems that these small marker chromosomes may increase the risk for nondisjunction and UPD [127].

Patients with PWS due to an ID should be tested for an IC deletion by a laboratory which is experienced in detecting them. The majority (approximately 85%) of those with an ID have a de novo epigenetic pathogenic variant (IIIb) and the recurrence risk is < 1% for this group. However, approximately 15% of those have ID with IC deletion (IIIa). In approximately half of these individuals, the IC deletion is familial and the recurrence risk is 50% for these families, as showed in Table 5. Therefore, fathers of children with an IC deletion should have DNA methylation and dosing analysis (or sequence analysis) to determine whether they carry the IC deletion.

**Table 5:** Risks to sibs of a proband with Prader-Willi syndrome by genetic mechanism

Molecular class	Genetic mechanism	Frequency of class (%)	Risk to sibs
I	Paternal deletion	65–75	< 1%
	Chromosome rearrangement	< 1	About 50%
II	mUPD	20–30	< 1%
	mUPD with predisposing parental translocation or marker chromosome	< 1	Almost 100% if mother has a 15;15 Robertsonian translocation
III	ID with IC deletion	< 0.5	About 50% if father also has an IC deletion
	ID without IC deletion	2	< 1%

ID, imprinting defect; IC, imprinting center; mUPD, maternal uniparental disomy

With rare exception, individuals with PWS do not reproduce. However, there were two reported female patients with genetically confirmed PWS who have had a child [112, 113]. No genetically confirmed males with PWS have been known to have fathered a child. The risk of the child of an affected individual depends on the etiology of the PWS and the gender of the affected individual. The offspring have a 50% risk of having AS, if the female proband has PWS with a deletion, due to the loss of function of the maternal copy of the *UBE3A* which is also located on the 15q11.2-q13.1. Almost all offspring of PWS patients with Robertsonian translocation are PWS or AS due to trisomy rescue or monosomy.

## Conclusions

The complex genetics, etiology, multiple phenotypes, and evolving natural history of PWS mean that a multidisciplinary professional, parental, societal, and environmental approach to the management is required with many challenges to reduce morbidity and mortality and improve quality of life. However, in recent years, there has been increasing appreciation and availability of important management strategies, which have already made significant improvements in the life of individuals with PWS. The management strategies include early diagnosis, evaluation and treatment by multidisciplinary teams, introduction of GH treatment, control of the food environment, and better understanding of the behavioral and psychiatric aspects. Whereas filling of the gaps in our understanding of the underlying science will translate and eventually guide clinical management of PWS (e.g. identification of genes and their link to particular phenotypes, genotype–phenotype correlations), several clinical and pathophysiological questions need to be urgently addressed to continue improvement in the care of patients with PWS.

## Data Availability

Data sharing not applicable to this article as no datasets were generated or analysed during the current study.
